# Gut microbiota shifts from onset to remission in immune checkpoint inhibitor-induced enterocolitis: a case report

**DOI:** 10.1186/s13099-024-00630-y

**Published:** 2024-07-04

**Authors:** Yuki Hirata, Yoshiki Tanaka, Haruka Yokota, Hiroshi Ohno, Koji Nishida, Hikaru Shimizu, Noboru Mizuta, Kei Nakazawa, Ryoji Koshiba, Kazuki Kakimoto, Takako Miyazaki, Shiro Nakamura, Hiroki Nishikawa

**Affiliations:** 1 Second Department of Internal Medicine, Osaka Medical and Pharmaceutical University, 2-7 Daigakumachi, Takatsuki, Osaka 569-8686 Japan; 2 Biofermin Pharmaceutical Co., Ltd., Kobe, Japan

**Keywords:** Immune-related adverse events, Enteritis, Microbiota, Immune checkpoint inhibitors

## Abstract

**Background:**

Immune checkpoint inhibitors (ICIs) are crucial in cancer treatment; however, they carry the risk of immune-related adverse events (irAEs), such as enteritis. Case presentation: This study investigated the role of the gut microbiota during the onset and remission of irAE enteritis in a patient with stage IV melanoma undergoing anti-PD-1 and anti-CTLA-4 therapy. Following commencement of ICI treatment, the patient developed severe diarrhea and was diagnosed with grade 3 irAE enteritis. Steroid and probiotic treatments provided swift symptom relief and remission, as confirmed by reduced fecal calprotectin levels and gastrointestinal imaging. Microbiota diversity analysis conducted via 16S rRNA gene sequencing identified a decrease in *Streptococcus* prevalence with improvement in enteritis symptoms. Conversely, genera *Fusobacterium*, *Faecalibacterium*, *Bacteroides*, *Prevotella*, and *Bifidobacterium* showed increased representation after remission. These genera are associated with anti-inflammatory properties and fibrous substrate degradation, aiding gut health. Immunological assessment demonstrated fluctuations in cytokine expression and the modulation of costimulatory molecules, aligning with therapeutic interventions and microbiota alterations.

**Conclusions:**

Our findings indicate a significant correlation between gut microbiota and immune responses in irAE enteritis. This underscores the potential utility of microbiome profiling in predicting irAE occurrence and in providing treatment strategies, thereby promoting a more comprehensive approach to managing the adverse effects of ICIs.

## Background

As immune checkpoint inhibitors (ICIs) have become increasingly used across various cancer types, the associated immune-related adverse events (irAEs) have garnered attention. Among these, irAE-induced colitis has been reported in approximately 5.7–9.1% of patients treated with anti-cytotoxic T-lymphocyte associated protein 4 (CTLA-4) antibodies and 0.7–1.6% of patients with anti-programmed cell death protein 1 (PD-1) antibodies. The incidence of colitis increases to 13.6% when these agents are used in combination [1]. Typically, colitis occurs after two–three administrations of ICIs or within 5–10 weeks of treatment initiation, although its onset can vary and it sometimes occurs after the first dose or during treatment interruption. The severity of symptoms of the irAE of colitis was standardized using the Common Terminology Criteria for Adverse Events grading, and steroids were recommended as the first-line treatment for irAE colitis.

However, the mechanisms underlying irAE colitis remain largely unknown. Unlike cytotoxic chemotherapies, ICIs do not directly affect the intestinal epithelia, suggesting a role of immune effector cells and the gut microbiota. Numerous studies have demonstrated the influence of gut microbiota composition on the antitumor efficacy of ICIs [2]. Additionally, fecal microbiota transplantation has been reported to be effective in patients with irAE colitis, suggesting a link between gut bacteria and irAE colitis [3]. Gut microbiome diversity has been associated with a decreased risk of irAE colitis [4]. However, studies directly observing the impact of ICIs on gut microbiota during irAE colitis onset are scarce.

This report provides a detailed investigation of the changes in gut microbiota from the onset to resolution of irAE colitis, highlighting the dynamic shifts within the microbial community.

### Case presentation

**Fig. 1:**
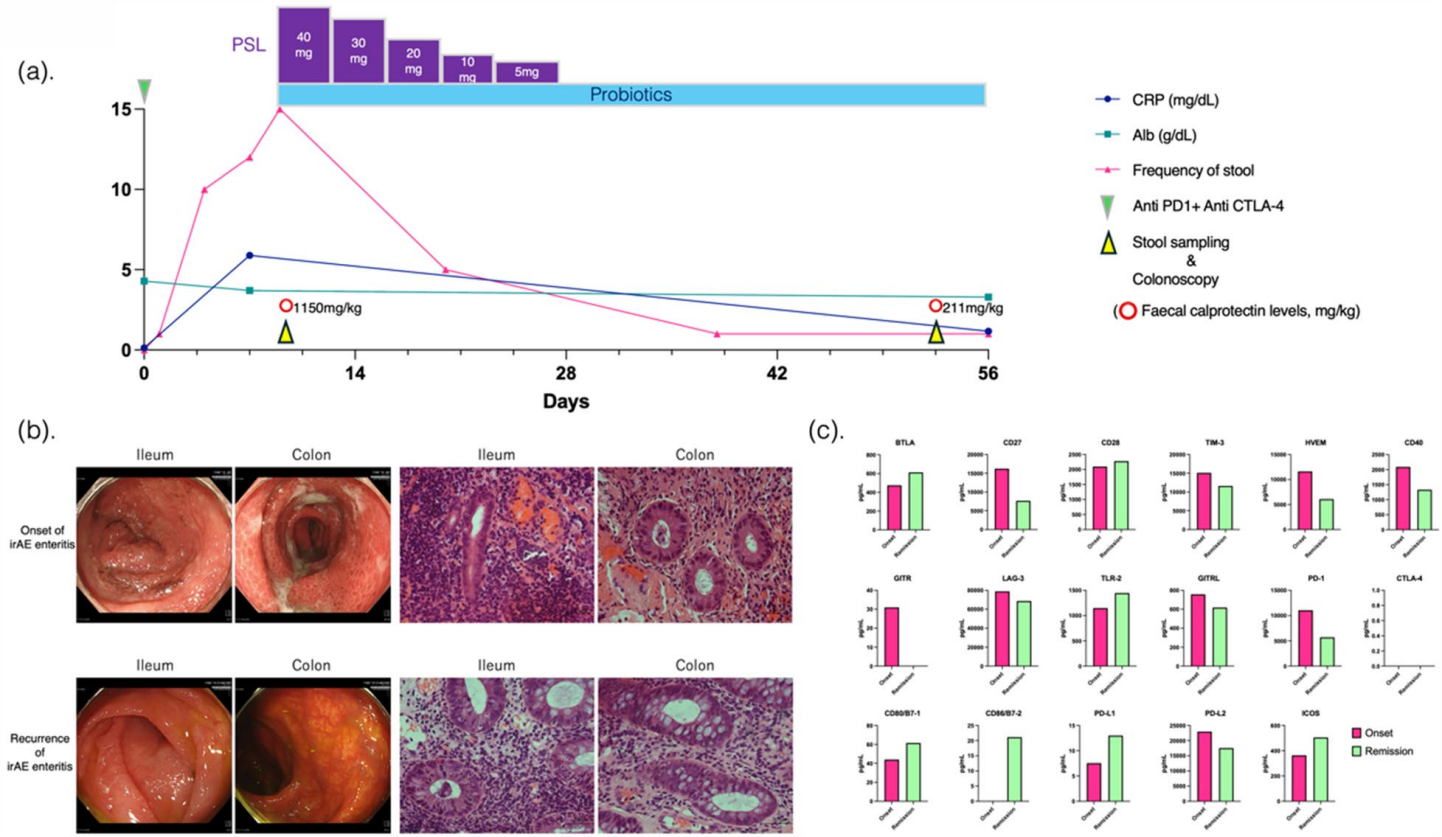
Clinical course of the patient after the first administration of immune checkpoint inhibitors (ICIs), with endoscopic and histopathological findings. **a** Clinical course: Patient's clinical timeline following the initial ICI administration, including onset and treatment of immune-related adverse event (irAE) enteritis, temporary remission, and recurrence after subsequent ICI treatment. **b** Initial irAE enteritis findings: Endoscopic and histopathological images of the small and large intestines during the first episode of irAE enteritis, showing the initial inflammatory response. **c** Recurrent irAE enteritis findings: Similar endoscopic and histopathological views during the recurrent episode of irAE enteritis

A 55-year-old male patient with stage IV melanoma was treated with systemic chemotherapy combined with anti-PD-1 antibody (nivolumab) and anti-CTLA-4 antibody (ipilimumab) treatment. Four days after initiation, he presented with acute symptoms of watery diarrhea, exceeding 10 episodes per day, and appetite loss (Fig. 1a). Elevated C-reactive protein levels (5.89 mg/dL) and abdominal computed tomography findings of ileal and colonic wall thickening, along with enlarged pericolic lymph nodes, heightened the suspicion of irAE enteritis, leading to referral for further diagnosis and management.

**Fig. 2:**
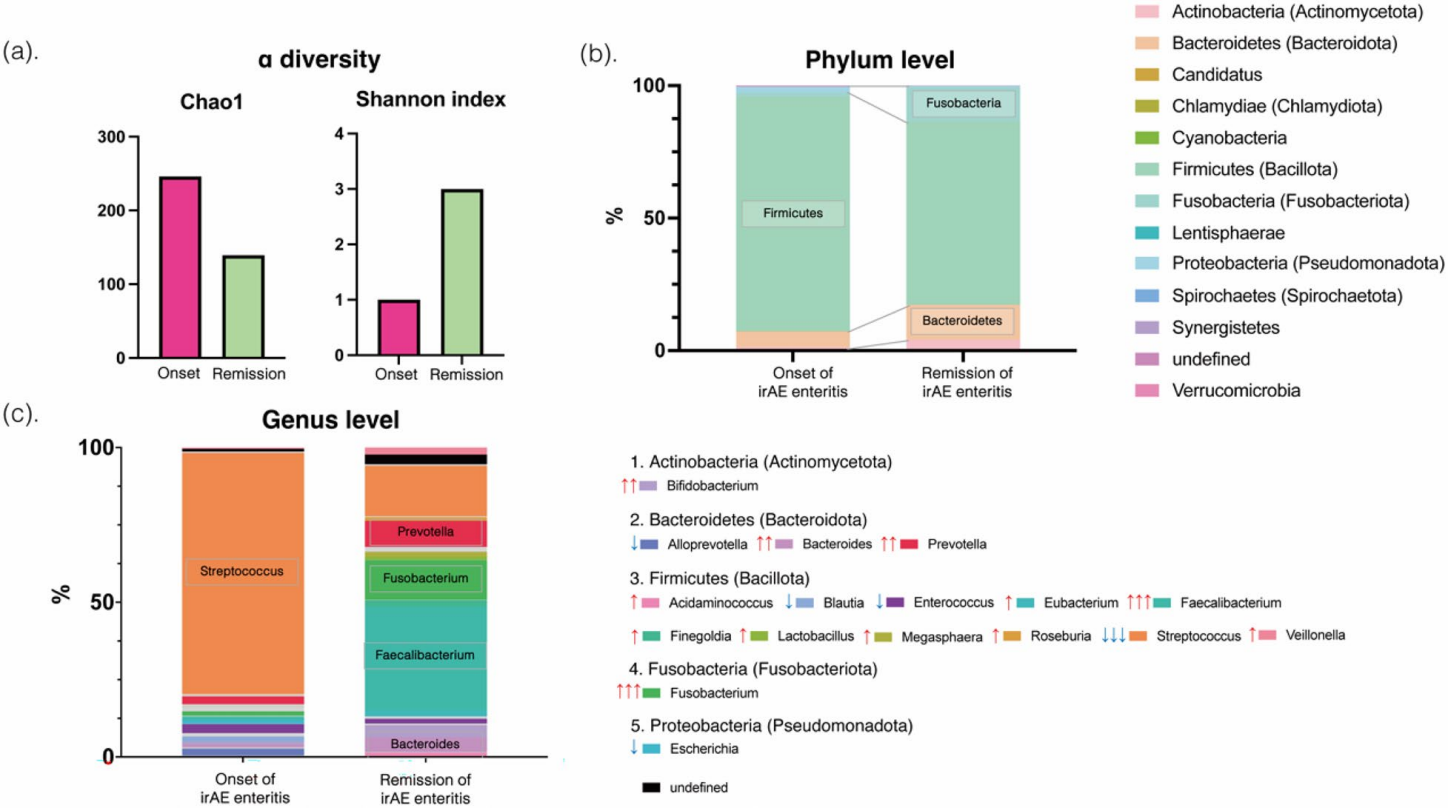
Changes in the gut microbiota during the onset and remission of immune-related adverse event (irAE) enteritis. **a** Diversity of gut microbiota: This panel compares the α diversity of the gut microbiota at the onset and during the remission of irAE enteritis, utilizing the Chao1 and Shannon indices for assessment. **b** This panel compares the gut microbiome at the phylum level between the onset and remission of irAE enteritis. **c** This panel illustrates a comparative analysis of the gut microbiome at the genus level during the onset and remission of irAE enteritis. Genera with a prevalence exceeding 1% are denoted in color. Downward arrows indicate a decrease in prevalence with the remission of enteritis, while upward arrows signify an increase. Changes in prevalence ranging from 3 to 10% are represented by double arrows, and changes exceeding 10% are depicted with triple arrows

Colonoscopy revealed continuous edematous and friable mucosa extending from the terminal ileum to the rectum with purulent mucus attachments, indicative of significant inflammation (Fig. 1b). Histological examination revealed lymphocytic infiltration and apoptotic bodies within the submucosa. Fecal calprotectin levels were markedly elevated at 1150 mg/kg, corroborating the observed inflammatory activity (Fig. 1a). Gut microbiota diversity was assessed by 16S rRNA gene sequencing of the stool samples (Fig. 2).

The patient was diagnosed with grade 3 irAE colitis and prompt treatment with prednisolone (40 mg/body) and probiotics (*Bifidobacterium bifidum*) was initiated. Symptoms rapidly ameliorated following the intervention, enabling tapering of outpatient prednisolone without recurrence. Subsequent colonoscopy and histological evaluations confirmed the resolution of enteritis, with fecal calprotectin levels reduced to 211 mg/kg (Fig. 1a, b).

Comparative enzyme-linked immunosorbent assay analysis of blood cytokines during irAE onset and resolution indicated the following immunological shifts: upregulation of B and T lymphocyte attenuator (BTLA) and toll-like receptor 2 (TLR-2), stable cluster of differentiation 28 (CD28) expression, and reduction in T-cell immunoglobulin and mucin-domain containing-3, lymphocyte-activation gene 3, herpes virus entry mediator, CD40, and glucocorticoid-induced tumor necrosis factor receptor-related protein (GITR). These changes illustrate modifications in the immune response mechanisms. Increased expression of the co-stimulatory molecules CD80/B7-1, CD86/B7-2, and PD-L1, with a decrease in PD-L2 expression, was observed. PD-1 levels decreased, aligning with the therapeutic targeting, whereas CTLA-4 levels remained minimally detectable throughout the treatment course (Fig. 1c).

Post-remission fecal sampling revealed significant changes in gut microbiota composition (Fig. 2). Alpha diversity decreased according to the Chao1 index but increased according to the Shannon index (Fig. 2a). At the phylum level, a decrease in *Firmicutes* and an increase in *Fusobacteria* and *Bacteroidetes* was observed along with the resolution of irAE colitis (Fig. 2b). Genus-level analysis revealed a substantial decline in *Streptococcus* species and an increase in *Fusobacterium*, *Faecalibacterium*, *Bacteroides*, *Prevotella*, and *Bifidobacterium* species post-remission (Fig. 2c).

## Discussion and conclusions

With the increasing use of ICIs, irAEs, such as enteritis, are becoming more frequent. While the mechanisms underlying irAE enteritis remain elusive, emerging studies have suggested the involvement of the gut microbiota. Our analysis focused on longitudinal changes in the gut microbiota of a patient who developed irAE enteritis and achieved remission through treatment.

Following the onset of irAE enteritis, the symptoms improved swiftly with steroid administration, and endoscopic and histological evaluations confirmed a reduction in inflammation. Fecal calprotectin has also been used as a marker of enteritis. Fecal calprotectin and lactoferrin are recognized as valuable markers of enteritis. The cutoff values for endoscopic remission and histological remission are reported to be less than 116 mg/kg and 80 mg/kg, respectively [5]. In our case, fecal calprotectin levels decreased from 1150 mg/kg at onset to 211 mg/kg at remission, paralleling the endoscopic findings and suggesting an improvement in enteritis.

We also examined cytokine profile changes from onset to remission of irAE enteritis. Initially, an increase in BTLA suggested enhanced inhibitory signaling, which might prevent the overactivation of CD8 + T cells. Along with enteritis improvement, a significant reduction in GITR levels indicated the potential recovery of regulatory T cells (Tregs), underlining GITR's role in the pathogenesis and resolution of irAE enteritis.

Moreover, a slight increase in TLR-2 expression corresponded with enteritis recovery, likely reflecting the immune system's response to gut microbiota diversity, and contributing to the resolution of inflammation.

The expression of PD-1 and CTLA-4, the key treatment targets, was compared during enteritis onset and remission. PD-1 levels decreased as enteritis recovered, likely because anti-PD-1 antibodies activated lymphocytes, causing PD-1 cleavage, and potentially increasing soluble PD-1 levels in the bloodstream, which then gradually declined. Conversely, CTLA-4 was minimally detectable throughout the study period.

Overall, these observations underscore the complex interplay among immune system activation, regulation, and microbial pattern recognition during the development and resolution of irAE enteritis.

Recent reports have increasingly suggested a role of the gut microbiome in irAEs, including enteritis. Gut microbial diversity is known to affect not only the exacerbation of irAE enteritis but also the responsiveness of tumors to ICI therapy [2]. Methods to evaluate microbial diversity within a single sample include the estimated total number of bacterial species, the Chao1 index, and the Shannon diversity index, which emphasize species richness and evenness. In this study, the Chao1 index decreased with the improvement in irAE enteritis, whereas the Shannon diversity index increased, suggesting that species evenness recovery is also crucial for recovery from irAE enteritis-induced dysbiosis.

Studies on the specific types of bacteria present before ICI administration have often investigated patients prone to developing irAEs. For instance, the abundance of microbes such as *Bacteroidetes* and *Bifidobacterium* has been associated with a reduced risk of gastrointestinal irAEs [6], whereas patients who developed gastrointestinal irAEs were reported to have an abundance of *Faecalibacterium* and *Firmicutes* prior to ICI administration. However, studies examining the changes in the gut microbiome before and after the onset of irAE enteritis are rare. A transition of the gut microbiome in pancreatic irAEs has been reported, with a decreased *Bacteroidetes*/*Firmicutes* ratio at the onset of irAE pancreatitis [7]. In our case, a very high prevalence of *Streptococcus* was observed at the onset of irAE enteritis, which decreased as the enteritis improved. In studies on patients with inflammatory bowel disease, the *Streptococcus* genus has been suggested to be an intestinal pathogen possibly originating from the oral cavity [8], and its increase has also been reported in radiation enteritis.

Other bacteria whose prevalence increased with improvement in enteritis included *Prevotella* and *Fusobacterium*, which can degrade fiber and produce short-chain fatty acids, and *Faecalibacterium* and *Bifidobacterium*, which have anti-inflammatory effects. The increase in the prevalence of these bacterial species is considered to have a positive effect on the normalization of the intestinal environment. In this case, the proportion of *Bacteroidetes* was low at the onset of irAE enteritis and increased upon remission of the enteritis with prednisolone treatment.

Within the *Bacteroidetes* species, *Bacteroides fragilis* has been reported to be involved in the downregulation of inflammatory Th17 cells [9], and *Bacteroides* has been reported to have a protective role against colitis [10]. Additionally, *Bacteroidetes* can decompose complex carbohydrates to produce simpler sugars and metabolites that can be utilized by other bacteria. *Fusobacterium* can grow using these metabolic products. Thus, the activity of *Bacteroides* may support an increase in *Fusobacterium*. *Bacteroides fragilis* produces polysaccharide A, which is involved in the regulation of the immune system and maintaining the balance of immune responses by activating T cells, particularly Tregs, in the gut. Thus, *Bacteroides* may play a significant role in the development of irAE enteritis.

Understanding gut microbiome and immunological changes at the onset of irAE enteritis is important for establishing future strategies for the prevention and risk assessment of irAE.

The findings from this case report demonstrate a significant shift in the gut microbiota profile during the occurrence of irAE enteritis, marked by an increase in the prevalence of specific bacteria and a recovery of diversity following treatment. These observations underscore the importance of the gut microbiome in the context of immune responses and irAE enteritis, suggesting that monitoring and potentially modulating the gut microbiota could serve as integral components of effective treatment strategies.

## Data Availability

Data not publicly available.

## Abbreviations

ICIs: Immune checkpoint inhibitors
irAEs: Immune-related adverse events
CTLA-4: Cytotoxic T-lymphocyte associated protein 4
PD-1: Programmed cell death protein 1
BTLA: B and T lymphocyte attenuator
TLR-2: Toll-like receptor 2
GITR: Glucocorticoid-induced tumor necrosis factor receptor-related protein
CD28: Cluster of differentiation 28
Tregs: Regulatory T cells
